# DOES SOCIAL ENGAGEMENT FOSTER GENERATIVITY AND GOOD CITIZENSHIP AMONG THE THIRD AGERS IN JAPAN?

**DOI:** 10.1093/geroni/igac059.2489

**Published:** 2022-12-20

**Authors:** Keiko Katagiri

**Affiliations:** Kobe University, Kobe, Hyogo, Japan

## Abstract

The labor force rate of those in their sixties have been increasing in Japan. Active Theory assumes that continuing activity is good for their well-being. Several researches also elucidate positive effects of work on older adults. Further, Japan has a mandatory retirement age of 60 years; though, persons can restart their work under contract labor. Katagiri proposed a social engagement model for third agers, which assumes that work for third agers cannot be the purpose in life and suggests three activities social participation, civic activities and work. This study examines the effect of the three activities on third agers. Random sampling survey was conducted in 2019 on people living in Tokyo and Hyogo prefecture. The response rate was 43.0% (N = 665, aged 60-74). Hierarchical multiple regressions were conducted to examine the effect of the three activities on generativity, sense of community, social contribution orientation, interest in politics by gender, and the robust positive effects of social and civic activities for male and civic activities for female. Though no effect of work existed, interaction effects of work and other activities were observed. Those working with social or civic activities had higher generativity. Among people with civic activity, working males showed higher sense of community while working females showed higher interest in politics. Males engaged only in work showed no such sense. The results suggest engaging only in work do not foster their development and engagement in social and civic activities are required to enrich their lives.

